# Effects of Probiotic Supplementation during Pregnancy on the Future Maternal Risk of Metabolic Syndrome

**DOI:** 10.3390/ijms23158253

**Published:** 2022-07-26

**Authors:** Aleksandra Obuchowska, Kamila Gorczyca, Arkadiusz Standyło, Karolina Obuchowska, Żaneta Kimber-Trojnar, Magdalena Wierzchowska-Opoka, Bożena Leszczyńska-Gorzelak

**Affiliations:** Chair and Department of Obstetrics and Perinatology, Medical University of Lublin, 20-090 Lublin, Poland; aobuchowska12@gmail.com (A.O.); karolinaobuchowska99@gmail.com (K.O.); zkimber@poczta.onet.pl (Ż.K.-T.); magdaopoka11@gmail.com (M.W.-O.); b.leszczynska@umlub.pl (B.L.-G.)

**Keywords:** probiotic supplementation, gestational diabetes mellitus, metabolic syndrome, preeclampsia, microbiome, obesity, pregnant women

## Abstract

Probiotics are live microorganisms that induce health benefits in the host. Taking probiotics is generally safe and well tolerated by pregnant women and their children. Consumption of probiotics can result in both prophylactic and therapeutic effects. In healthy adult humans, the gut microbiome is stable at the level of the dominant taxa: *Bacteroidetes*, *Firmicutes* and *Actinobacteria,* and has a higher presence of *Verrucomicrobia.* During pregnancy, an increase in the number of *Proteobacteria* and *Actinobacteria phyla* and a decrease in the beneficial species *Roseburia intestinalis* and *Faecalibacterium prausnitzii* are observed. Pregnancy is a “window” to the mother’s future health. The aim of this paper is to review studies assessing the potentially beneficial effects of probiotics in preventing the development of diseases that appear during pregnancy, which are currently considered as risk factors for the development of metabolic syndrome, and consequently, reducing the risk of developing maternal metabolic syndrome in the future. The use of probiotics in gestational diabetes mellitus, preeclampsia and excessive gestational weight gain is reviewed. Probiotics are a relatively new intervention that can prevent the development of these disorders during pregnancy, and thus, would reduce the risk of metabolic syndrome resulting from these disorders in the mother’s future.

## 1. Introduction

Probiotics are live microorganisms that induce health benefits to the host [1]. Prebiotics, such as inulin and fructooligosaccharides, are nondigestible food components that promote the growth of beneficial bacteria in the colon [2], whereas synbiotics are a mixture of live microorganisms with substrates that are selectively utilized by host which can provide even more benefits than prebiotics alone [3].

Recommendations regarding probiotics are complex and varied by country. There is a distinction between probiotics found in food and probiotics that are pharmaceuticals. The scientific criteria regarding the health benefits of probiotics are also becoming increasingly stringent [4]. Taking probiotics is generally safe and well tolerated by pregnant women and their children [5,6,7,8,9]. Nevertheless, side effects such as systemic infections, mild gastrointestinal disturbances and skin complications have been reported [5,10]. According to a study by Homayouni et al., food is a better carrier of probiotics than supplements [11]. Consumption of probiotics can result in both prophylactic and therapeutic effects. They may help to prevent infections, decrease cholesterol levels, promote the synthesis of vitamins and cytokines, and inhibit cancer progression [12]. Probiotics can normalize the composition of the microbiota of the gastrointestinal tract, genitourinary system, and prevent antibiotic-associated diarrhea and travelers’ diarrhea [13,14]. The intestinal microflora can be affected by various factors, and the use of probiotics during an infection or antibiotic therapy can improve it [15].

The human gut, mainly in the distal colon, contains approximately 100 trillion gut microflora, including bacteria, archaea, viruses, and eukaryotic microbes [16,17]. The gut microbiota maintains interaction with the host through digestion, metabolism, nutrient extraction, vitamin synthesis, and pathogen protection [17,18,19,20]. Moreover, it has been found that the role of the human microbiome goes beyond the digestive tract, and the brain-gut-microbiota axis has been described [21,22]. It is generally accepted that in healthy adult humans, the gut microbiome is stable at the level of the dominant taxa: *Bacteroidetes, Firmicutes* and *Actinobacteria* and a higher presence of *Verrucomicrobia* (*Akkermansia muciniphila*) [21,23,24]. A healthy gut microflora was linked to an increase in the diversity and abundance of *Bacteroidetes*, and a higher ratio of *Akkermansia muciniphila* to *Ruminococcus gnavus* [21].

During pregnancy, an increase in the number of *Proteobacteria* and *Actinobacteria phyla* and a decrease in the beneficial species *Roseburia intestinalis* and *Faecalibacterium prausnitzii* were observed [25,26]. Changes in the composition of the microbiota in pregnancy may be associated with an increase in adipose mass, blood glucose levels, insulin resistance (IR), and the circulation of pro-inflammatory cytokines in the pregnant woman [27].

During pregnancy, women undergo significant anatomical and physiological changes related to the development of the fetus and the preparation for delivery. The diseases that appear during pregnancy usually concern endothelial dysfunction and endocrine disorders. These diseases put the mother at high risk of developing diseases such as type 2 diabetes mellitus (T2DM), cardiovascular disease (CVD) and possibly the development of the metabolic syndrome (MS) as a consequence.

MS is defined as a group of related risk factors that increase the risk of overall mortality and morbidity, as well as the cost and burden of healthcare. MS is an independent risk factor for CVD including microvascular dysfunction, coronary atherosclerosis and calcification, cardiac dysfunction, myocardial infarction, and heart failure [28,29]. The criteria for diagnosing MS in women vary depending on the recommendation, and any three of the five criteria constitute a diagnosis of MS [30,31] (Table 1).

The aim of the research was to review studies on the assessment of the potentially beneficial effects of probiotics in preventing the development of diseases that appear during pregnancy, which are currently considered as risk factors for the development of MS, and consequently, the reduction of the risk of developing maternal MS in the future. The use of probiotics in gestational diabetes mellitus (GDM), preeclampsia (PE) and excessive gestational weight gain (EGWG) has been reviewed.

## 2. Pregnancy as a Window for the Development of Metabolic Diseases

Pregnancy is a “window” to the mother’s future health. We focused on the diseases occurring during gestation that have implications for long-term maternal complications and, therefore, their programming towards metabolic syndrome.

The scientific literature defines GDM as a state of hyperglycemia developing in pregnancy as a result of IR or reduced insulin production, which resolves following delivery [32,33,34,35,36]. GDM is the most common metabolic disease during pregnancy and it can affect 3–31% of women. In Europe, the incidence of GDM during pregnancy ranges from 2 to 6% [33,37,38,39,40,41]. The prevalence is particularly increased in high-income countries, paralleling excessive weight and obesity, multiple gestations, and delayed childbearing age [42].

Due to placental hormones such as human placental lactogen, progesterone, cortisol, growth hormone, and prolactin, pregnancy is linked to physiological IR, especially in the second and third trimester of pregnancy [43,44]. Recently, the role of leptin stimulating IR and the inhibitory role of adiponectin have been emphasized [45,46,47,48,49,50]. The pathogenesis of GDM also takes into account the participation of less known molecules such as galectins, growth differentiation factor-15, chemerin, omentin-1, osteocalcin, resistin, visfatin, vaspin, irisin, apelin, Fatty Acid-Binding Protein 4 (FABP4), fibroblast growth factor 21, lipocalin-2, fetuin-A and zonulin [43,44,46,51,52,53,54,55]. IR is seen in GDM patients, as well as in impaired insulin secretion due to a defect in pancreatic-cell function [33,37,56]. Chronic inflammatory reaction increases IR, disrupts the function of pancreatic β cells and insulin secretion. Vitamin D deficiency can also worsen the function of β cells in the pancreas [57,58,59,60].

It is observed that IR rises distinctly in pregnant women with pre-pregnancy obesity and excessive weight compared with those with a healthy pre-pregnancy weight [61]. It is well known that a key factor in limiting the action of insulin is the increased inflammatory response associated with excess body fat [62,63,64]. The risk factors for GDM are pre-pregnancy obesity and excess weight, EGWG, a family history of T2DM, advanced maternal age, multiparity, GDM during previous pregnancies, giving birth to a baby with a birth weight greater than 4000 g in previous pregnancies, and polycystic ovary syndrome [65,66]. It should be noted that diabetes often coexists with obesity.

GDM is gaining increasing attention from researchers due to the serious risks and adverse health effects for the mother and her offspring. The main health consequences include an increased risk of PE, macrosomia, polyhydramnios, stillbirth and the development of metabolic diseases in the offspring in the future [67,68,69,70,71,72,73].

Women with a history of GDM have an increased risk of recurrence of GDM in subsequent pregnancies. Evidence suggests that GDM is a precursor to T2DM in predisposed women [74]. In women with GDM, the risk of developing T2DM is 7–10 times higher than in women without GDM [75,76,77,78,79]. Furthermore, the development of T2DM in patients with GDM has been associated with waist circumference, BMI, early gestational age at the time of diagnosis and gestational insulin use [80,81]. Women with GDM are at increased risk of developing CVD postpartum, which in combination with T2DM, suggests an increased incidence of MS in the future [79,82]. GDM cannot be absolutely prevented; however, early diagnosis and prompt therapy meaningfully improve the development of the fetus, the pregnancy course, delivery, and the postpartum period, as well as the development of civilization diseases in the future. Furthermore, GDM is a long-term risk factor for the development of T2DM, CVD, MS, malignant neoplasms, maternal ophthalmic, mental and renal diseases and non-alcoholic fatty liver disease [69,75,83].

MS comprises central obesity, increased plasma glucose levels, dyslipidemia and hypertension, and its incidence is increasing due to changes in lifestyle and an incorrect diet in recent years. Between 28% and 60% of women with GDM will develop MS later in life, excluding women with GDM who already have parallel MS (with central obesity, hypertension and high level of TG and/or low level of HDL cholesterol) [80,84,85,86]. In women with GDM, the risk of developing MS is 3–6 times higher than in women with normoglycemia during pregnancy [27,29,30]. Women with GDM who deliver large for gestational age (LGA) neonates have a higher prevalence of MS later in life than women with normoglycemia and LGA offspring [87].

In recent decades, the worldwide prevalence of obesity and the related metabolic disorders has increased dramatically. Obese or overweight people face a 10-fold increased risk of developing MS [88]. Current and pregestational obesity and excess weight were found to be important predictors of MS in some studies, especially when combined with a fasting glycemia [80,84,89]. Compared with women with manifest diabetes, the rate of MS is delayed by more than ten years in women with glucose intolerance [57]. Obese people’s microbiomes are physically and functionally diverse from their healthier counterparts, according to research [90]. Ferrer et al. found that in the gut of obese people, *Firmicutes* (94.6%) were more numerous than *Bacteroidetes* (3.2%), whereas the gut of lean people showed a change towards increased *Bacteroidetes* (18.9%) [91]. Additionally, the intestinal microbiome of an obese person is less diverse than that of lean person [92,93]. This strongly suggests the possibility of using the microbiome in the therapy of obesity.

The extended low-grade inflammation which accompanies MS and obesity is associated with the growth of adipose tissue. Currently, adipose tissue is considered as a potent immune and endocrine organ [94]. It consists of endothelial cells, preadipocytes, adipocytes, fibroblasts, and immune cells, including mainly T cells, B cells, macrophages, and neutrophils [95,96,97]. Excessive caloric intake, body weight and diet control the cellular composition of adipose tissue, especially the composition of the immune cells. In response to these stimuli, immune cells of the stromal vascular fraction change from anti-inflammatory subtypes to more pro-inflammatory subtypes [98]. It is known that adipokines secreted by adipose tissue are involved in inflammatory processes. Patients with pre-existing obesity have a greater risk of subclinical inflammation, IR and endothelial dysfunction leading to the development of MS [80]. Some evidence suggests that MS and GDM may have a common genetic basis [99]. Furthermore, GDM and MS appear to have similar pathophysiologic pathways, as evidenced by a link between the risk gene variants TCF7L2 and FTO [100].

Women at a higher risk of developing MS should be identified using antenatal glucose measurements and BMI [85]. Effective treatment of GDM may be a way to avoid the development of cardiovascular complications in the future [85]. The aim of GDM therapy is to maintain normoglycemia and prevent excessive weight gain to reduce maternal and fetal complications. Exercise and nutritional therapy are examples of lifestyle changes. To avoid postprandial hyperglycemia and reduce IR, caloric restriction combined with a low glycemic index diet is recommended [101]. It is important to look for other methods to prevent or support the treatment of the GDM in order to prevent the onset of MS in the future.

## 3. Therapeutic Applications of Probiotics

### 3.1. The Use of Probiotics in GDM and EGWG

Preventing GDM, rather than treating it, can have a number of benefits, both health and economic. Homayouni et al. suggest that probiotics are a relatively new intervention that can lower glucose levels, prevent GDM, and reduce maternal and fetal complications resulting from it [102]. Several studies have shown that the gut microflora is significantly altered in women with GDM and is similar to that of adults with T2DM [103,104,105]. An increased number of gram-negative bacteria, such as *Parabacteroides*, *Prevotella*, *Haemophilus*, and *Desulfovibrio*, has been found in the intestines of GDM patients [104,106,107,108]. However, a study by Mokkala et al. showed that the presence or absence of a specific bacterial species or function did not predict the onset of GDM, nor did it differ depending on the severity of GDM [109], although in the group of women with GDM, a higher number of *Ruminococcus obeum* was found in late pregnancy [109].

The roles of probiotics in modulating the composition of the intestinal microbiota and reducing the adherence of pathobionts, regulating the permeability of the intestinal epithelium and reducing the inflammatory process has been observed [77]. A randomized study of probiotics (*Lactobacillus rhamnosus GG* and *Bifidobacterium lactis*) showed a reduction of more than 60% in GDM, with an incidence of 13% in the probiotic group compared with 34% in the control group [110]. However, some studies found no significant differences between the probiotic and placebo groups in terms of glycemic control and antioxidant capability [111,112]. Nevertheless, the study by Sahhaf Ebrahimi et al. found that the use of probiotic yogurt improved blood glucose levels as well as glycated hemoglobin (HbA1c) levels [112]. Probiotic supplementation may improve blood glucose control during the third trimester, according to a study conducted in healthy pregnant women [113]. Supplementing with probiotics reduced fasting plasma glucose, serum insulin levels, and IR while increasing insulin sensitivity [7,114,115]. However, other studies have shown no benefit of taking probiotics in reducing the risk of GDM [116,117,118,119,120] or improving glucose metabolism in overweight and obese women [109,119,120]. Studies in women with GDM found no significant differences in fasting glucose levels between a group receiving supplementation of *Lactobacillus salivarius* and a placebo group [121]. In contrast, a reduction in insulin resistance (HOMA-IR) and β-cell function (HOMA-B) was observed after probiotic administration [7,115,122,123,124]. Other studies have shown that taking probiotics containing *Lactobacillus acidophilus*, *Lactobacillus casei* and *Bifidobacterium bifidum* in GDM patients had a beneficial effect on glycemic control, TG levels and very low-density lipoprotein (VLDL) [114,122].

The potential positive effects of using probiotics may also result from other mechanisms. Reduction of oxidative stress and increased secretion of incretin are considered as potential mechanisms by which probiotics improve glucose metabolism [125,126,127,128]. *Bifidobacterium* and *Lactobacillus* are among the most common, non-pathogenic living microorganisms used as probiotics [129]. They have been identified as probiotics that reduce systemic inflammation, regulate immune function, improve intestinal mucosa permeability, and ultimately reduce IR [130,131,132,133]. *Bifidobacteria* are commensals of humans and animals, they belong to the phylum of *Actinobacteria* [4]. *Lactic acid bacteria* are gram-positive bacteria that are traditionally used in the production of yogurt and other fermented dairy products [4]. Similar results were presented by Badehnoosh et al., who showed that taking a probiotic capsule containing *Lactobacillus acidophilus*, *Lactobacillus casei*, and *Bifidobacterium bifidum* (2 × 109 CFU/g each) for 6 weeks improved the glycemic response and markers of inflammation but did not affect pregnancy outcomes [134].

In addition, recent studies indicate that plasma vitamin C levels negatively correlate with the development of MS. It has been suggested that vitamin C supplementation may help reduce oxidative stress and postpone the chronic inflammation associated with the development of MS.

The relative abundance of short-chain fatty acids (SCFA) producing bacteria from the genera *Faecalibacterium*, *Ruminococcus*, *Roseburia*, *Coprococcus*, *Akkermansia*, *Phascolarctobacterium*, and *Eubacterium* was found to be lower in GDM women, obese and T2DM [104,105,106,108,135,136,137,138,139,140,141]. SCFA affect the activity of cells of the immune system, as well as their migration to the site of inflammation, showing a significant anti-inflammatory potential [142]. The results of many animal studies show that supplementation with the probiotic *Lactobacillus* spp. induces the production of SCFA by modulating the intestinal microbiome [141,143,144,145,146]. Manipulating the composition of the gut microbiome, and thus the level of SCFA, may prove to be a promising method in the treatment of inflammatory diseases [142].

In the peripheral blood of patients with GDM, probiotic intake increases the expression of peroxisome proliferator-activated receptor gamma (PPAR-γ), transform growth factor beta (TGF-β), and vascular endothelial growth factor (VEGF) and decreases the expression of tumor necrosis factor alpha (TNF-α) [114]. This suggests that probiotics alleviate IR and chronic inflammation through the PPAR pathway [147].

The supplementation of probiotics also results in a considerable decrease in plasma malondialdehyde (MDA) and a significant increase in plasma nitric oxide (NO) and total antioxidant capacity [114]. MDA has cytotoxic, mutagenic and carcinogenic properties [148]. It can also inhibit enzymes related to the cell’s defence against oxidative stress [148]. NO is the main factor in endothelial cells responsible for maintaining vascular homeostasis [149].

The studies found that there was no significant difference in reducing GWG in overweight or obese pregnant women between the probiotic group (*Lactobacillus rhamnosus* and *Bifidobacterium lactis*) and the placebo group [119,150,151].

### 3.2. The Use of Probiotics in PE

PE is a complicated disorder in pregnancy which occurs after 20 weeks of gestation and affects 2–8% of all pregnancies in the world [152,153,154]. PE is a vascular pregnancy complication with high fetal and maternal mortality and morbidity rates [155,156]. The presence of PE in the past increases the risk of hypertension, ischemic heart disease, venous thromboembolism, kidney disease and CVD, including myocardial infarction, stroke, and heart failure [152,157,158,159,160,161]. PE is associated with a 4-fold increased risk of future stroke [162]. PE is associated with a 2–7 times higher risk of CVD, especially if PE occurs before 34 weeks of gestation [157,163,164,165,166]. Research has shown a significant relationship between the occurrence of PE and MS later in life [167,168,169,170,171,172]. PE is connected to MS risk factors, which are modifiable CVD risk factors [164,173,174]. According to research by Heidema et al., obesity, IR, and arterial hypertension are all common within the first year following a pregnancy affected by PE [159]. Moreover, Hooijschuur et al. showed that the incidence of MS was higher in the subgroup of women with PE and small-for-gestational-age (SGA) neonate than in women without SGA neonate [164]. It is important to look for new interventions that can reduce the risk of developing PE and consequently, complications for the mother and offspring.

Brantsæter et al. showed that consumption of milk-based probiotic products was associated with a reduced risk of overall PE, with the association most prominent in severe PE [175]. Probiotic consumption has been linked to lower blood pressure in non-pregnant women, according to clinical intervention trials using milk-based probiotic supplements [176,177]. The results of a study by Nordqvist et al. showed that consumption of probiotic milk in late pregnancy was associated with a reduced risk of PE, and consumption in early pregnancy was associated with a reduced risk of preterm delivery [178]. On the other hand, a 2018 Cochrane review of maternal oral probiotic supplementation did not find appreciable benefit or harm to neonates as a result of supplementation of pregnant women at low risk of preterm birth [179]. Furthermore, the study by Yeganegi et al. showed that *Lactobacillus rhamnosus* influences the lipopolysaccharide (LPS) response in placental trophoblast cells, which may affect the inflammatory response important in the pathophysiology of PE development [180]. However, a 2021 Cochrane review showed that probiotics can be harmful by increasing the risk of hypertensive disorders in pregnancy including PE [116]. The authors report that the best variables to predict the occurrence of MS in women with prior PE are early-onset PE, SGA newborn and measurement of systolic blood pressure [164].

### 3.3. The Use of Probiotics in Obesity and Lipid Disorders

The influence of obesity on the development of the MS is unquestionable, and the prevention of excessive weight gain may reduce the risk of complications in the future. Diets high in saturated fat and fructose affect the composition of the gut flora [181]. The resulting dysbiosis leads to a cascade of events including increased intestinal barrier permeability, bacterial translocation, and activation of hepatic receptor-induced inflammation [182]. One proposed mechanism pertains to the production of endogenous alcohol and acetaldehyde [183,184,185].

Probiotics containing *Lactobacillus paracasei* can impact adipose tissue mass by modulating the activity of angiopoietin-like protein 4, a circulating lipoprotein lipase (LPL) inhibitor that controls TG deposition into adipocytes, which can help prevent obesity and metabolic disorders [186].

The beneficial effect of weight reduction was obtained during *Lactobacillus gasseri* (*SBT2055*) supplementation in overweight and obese people [187,188]. Daily consumption of 200 g of yogurt containing *Lactobacillus gasseri* (108 CFU/g) for 12 weeks significantly reduced abdominal obesity. BMI, waist and hip circumferences, and body fat mass were also significantly decreased from the baseline, but discontinuation of the probiotic for 4 weeks weakened these effects [187]. Additionally, in studies conducted by Ilmonen et al., supplementation with probiotics *Lactobacillus rhamnosus GG* and *Bifidobacterium lactis* showed a reduction in maternal central adiposity at 6 months postpartum [189].

*Lactobacillus plantarum* (*PL62*) as a probiotic has been shown in two mouse trials to produce conjugated linoleic acid (CLA), which has been linked to weight loss [190,191]. In a study on mice by Bagarolli et al. the effects of probiotics (*Lactobacillus rhamnosus*, *Lactobacillus acidophilus* and *Bifidobacterium bifidum*) on the intestinal microflora, changes in insulin permeability, sensitivity and signaling in a high-fat diet (HFD) were investigated [192,193]. Probiotic-treated mice receiving a HFD gained significantly less weight and had reduced food consumption and also reduced fasting blood glucose and serum insulin compared with animals that did not receive probiotics [28]. The administration of probiotics in mice fed HFD improved leptin sensitivity [192,194,195]. The study showed that the administration of probiotics to obese animals was able to reduce Toll-like receptor 4 (TLR4) activation, downstream c-Jun N-terminal kinases (JNK) phosphorylation and the subsequent insulin receptor substrate-1 (IRS1Ser307) phosphorylation [192]. The probiotic administration had no effect on TLR4 signalling in mice with a normal body weight [192]. TLR4 is a LPS receptor that plays an important role in the regulation of immunological responses to infection [196]. As one of the components of the outer membrane of Gram-negative bacteria, LPS is considered an endotoxin that may contribute to the development of inflammation and IR [197,198]. The relative levels of TNF-α and Interleukin 6 (IL-6) transcripts in the liver, muscle and blood of probiotic-treated mice were significantly lower than in the control group [192]. When compared to untreated mice, treatment with probiotics boosted the predominance of *Firmicutes* and *Actinobacteria* while decreasing the presence of *Bacteroidetes* [192]. Obese animals also had a higher frequency of *Bacteroidetes* and a lower number of *Firmicutes* and *Actinobacteria* [192]. In obese animals, probiotic therapy resulted in the continuing presence of *Bacteroidetes*, an increase in the prevalence of *Actinobacteria*, and a decrease in *Firmicutes* compared with the control group [192]. An increased number of bacteria from the taxonomic family *Lachnospiraceae* has been associated with the development of diabetes in obese mice [18,199]. Probiotic administration has beneficial effects on the host, including increased adipose tissue lipolysis. Anorexigenic peptides, such as glucagon-like peptide-1 (GLP-1) and peptide YY (PYY), are secreted as a result, leading to an enhanced glucose tolerance and greater energy utilization [200,201,202]. In addition, treatment with probiotics decreased the expression of the main molecules involved in intestinal microflora inflammation and bacterial translocation, nucleotide-binding oligomerization domain-containing protein 1 (NOD-1) and CD-14 [192]. Treatment with probiotics significantly reduced the adipocyte surface area in obese mice, but the values were still outside the norm [192].

A study by Fabersani et al. showed that the *Lactobacillus fermentum* (*CRL1446*) strain induced a decrease in the production of leptin in adipose tissue [94]. In animal studies, this strain has antioxidant, hypoglycemic and hypocholesterolemic properties, making it an interesting alternative in the treatment of obesity, which is characterized by elevated levels of leptin in the serum [203,204].

A study by Le Barz et al. showed that mice fed the high-fat high-sucrose (HFHS) diet and treated with *Lactobacillus plantarum* (*Lb38*), *Lactobacillus rhamnosus* (*Lbl02*) or *Bifidobacterium animalis* ssp. *lactis* (*Bfl41*) exhibited a significant decrease in weight gain compared with the control group [205]. *Lbl02* and *Bf141* significantly reduced visceral obesity, and improved insulin sensitivity and glucose tolerance in HFHS-fed mice [205]. The applied probiotics reduced the concentration of pro-inflammatory chemokines (MCP-1 and RANTES) [205]. Long-term administration of Lbl02 and Lb38 significantly lowered the plasma leptin concentration compared with the control group [205]. Lbl02 and Bfl41 showed a tendency to reduce HFHS-induced lipid accumulation in the liver [205]. The gene expression of zonula occludens 1 (zo-1) and occludin, which code for key tight-junction proteins controlling epithelial integrity, was greatly elevated after Lbl02 therapy. Mucins 2 and 3 (muc2 and -3) gene expression was also raised following Lbl02 therapy, implying that the gut barrier could be strengthened by increasing mucus-layer thickness [205].

Thiennimitr et al. showed that probiotics *Lactobacillus paracasei* (*HII01*), prebiotic xylooligosaccharide (XOS), and synbiotics reduce intestinal dysbiosis and enteritis, leading to improved metabolic dysfunction in obese insulin-resistant rats [206].

Numerous studies have shown that dysbiosis, small intestinal bacterial overgrowth and increased intestinal permeability have a role in the development of non-alcoholic fatty liver disease (NAFLD) and non-alcoholic steatohepatitis (NASH), both of which are closely linked to MS [207,208,209,210]. Dysbiosis was manifested in these disorders by an increase in *Enterobacteriaceae* and *Proteobacteria* and a decrease in *Bacteroidetes* [208]. The specific composition of gut microbiota may play a role in both the inflammatory and fibrosis responses in patients with NAFLD [211]. In patients with NASH, an increase in the numbers of *Bacteroides* has been shown, and a higher degree of fibrosis has been observed in patients with an increased amount of *Ruminococcus* [212,213].

A meta-analysis showed that treatment with *Lactobacillus acidophilus*, a mixture of *Lactobacillus acidophilus* and *Bifidobacterium lactis*, and *Lactobacillus plantarum* for 3 to 12 weeks lowered total and low-density lipoprotein (LDL) cholesterol concentrations compared with a placebo [214,215,216]. On the other hand, no effect of *Lactobacillus helveticus* and *Enterococcus faecium* on cholesterol concentrations was demonstrated [214]. Studies found no significant differences in LDL cholesterol in women with GDM between a group with supplementation of *Lactobacillus salivarius* and a placebo group [120]. In a research review by Okesene-Gafa et al. it was observed that taking probiotics may be associated with a slight reduction in TG and total cholesterol [115]. In a study by Babadi et al., probiotic capsules containing *Lactobacillus acidophilus*, *Lactobacillus casei*, *Bifidobacterium bifidum* and *Lactobacillus fermentum* reduced the concentration of TG, VLDL cholesterol, and the total/HDL cholesterol ratio while increasing HDL cholesterol levels [114].

NAFLD is strongly associated with obesity and thus closely related to the elements of MS (abdominal fat distribution, IR, diabetes, dyslipidemia, and hypertension) [217,218,219,220,221,222]. Preventing the development of NAFLD and NASH may contribute to reducing the risk of developing MS in the future.

The table below summarizes the studies conducted in pregnant women on the effects of probiotics on metabolic disorders (Table 2).

### 3.4. Probiotics and the Prevention of the Development of MS

The studies suggest that the intestinal microbiota is a key player in the development of a chronic low-grade inflammatory state associated with MS [206]. The relationship between gut microbiota and the onset of metabolic inflammation related to obesity, IR and T2DM has been demonstrated [223,224]. Metabolic endotoxemia, which is caused primarily by the Gram-negative bacterial membrane component—LPS, is a critical event in the development of these conditions [224,225]. Through metabolic pathways, LPS leads to pro-inflammatory changes (increases in TNF-α, IL-1β and IL-6, leptin and resistin, plasminogen activator inhibitor-1 and C-reactive protein) and induces IR [226].

It has been noted that specific probiotics may, apart from their immunomodulatory and metabolic effects, modulate the intestinal microflora [12]. For these reasons, probiotics may play an important role in immunomodulation to help prevent the low-grade chronic inflammation associated with MS [4,94,226].

Inflammatory reactions in the gut can occur through activation of the TLR pathway, degradation of the IĸB kinase, and release of nuclear-kappa B factor (NF-ĸB), which activates the pro-inflammatory cascade [227,228]. Several probiotic strains such as *Lactobacillus rhamnosus* or *Lactobacillus casei* have been shown to be effective in preventing IĸB breakdown and thus reducing the release of pro-inflammatory molecules [229,230].

SCFAs such as acetate, propionate, and butyrate can be catabolized by probiotics from complex polysaccharides from the diet [231]. These substances are thought to help with metabolic disorders associated with MS. SCFAs show significant anti-inflammatory potential by reducing IR, and increasing the secretion of the protective GLP-1, which stimulates insulin release and improves β-cell function [142]. A study by Yadav et al. showed that acetate can suppress insulin signaling in adipocytes, inhibiting fat accumulation in adipose tissue [126].

It is important not only to take probiotics, but also other substances which affect probiotics bioavailability. Therefore, further work should pay attention to additional aspects including plasma concentrations of substances such as beta glucans and curcuminoids in pregnant women, which may affect probiotic absorption and increase the well-being of the gut microflora [232,233]. Vitamin C taken with probiotics may multiply their beneficial effect by reducing oxidative stress and postponing the chronic inflammation associated with the development of chronic diseases, including MS [234]. In addition, a proper diet rich in whole grains should be beneficial and could impact the microbiota profile in these patients [232].

Our study has some limitations. This work is not a systematic review and publications covering animal studies were included.

## 4. Conclusions

Pregnancy is said to be “a window to future health” as the occurrence of characteristic complications during pregnancy may trigger a vascular or metabolic risk of developing civilization diseases in the future life of the mother.

**Figure 1:**
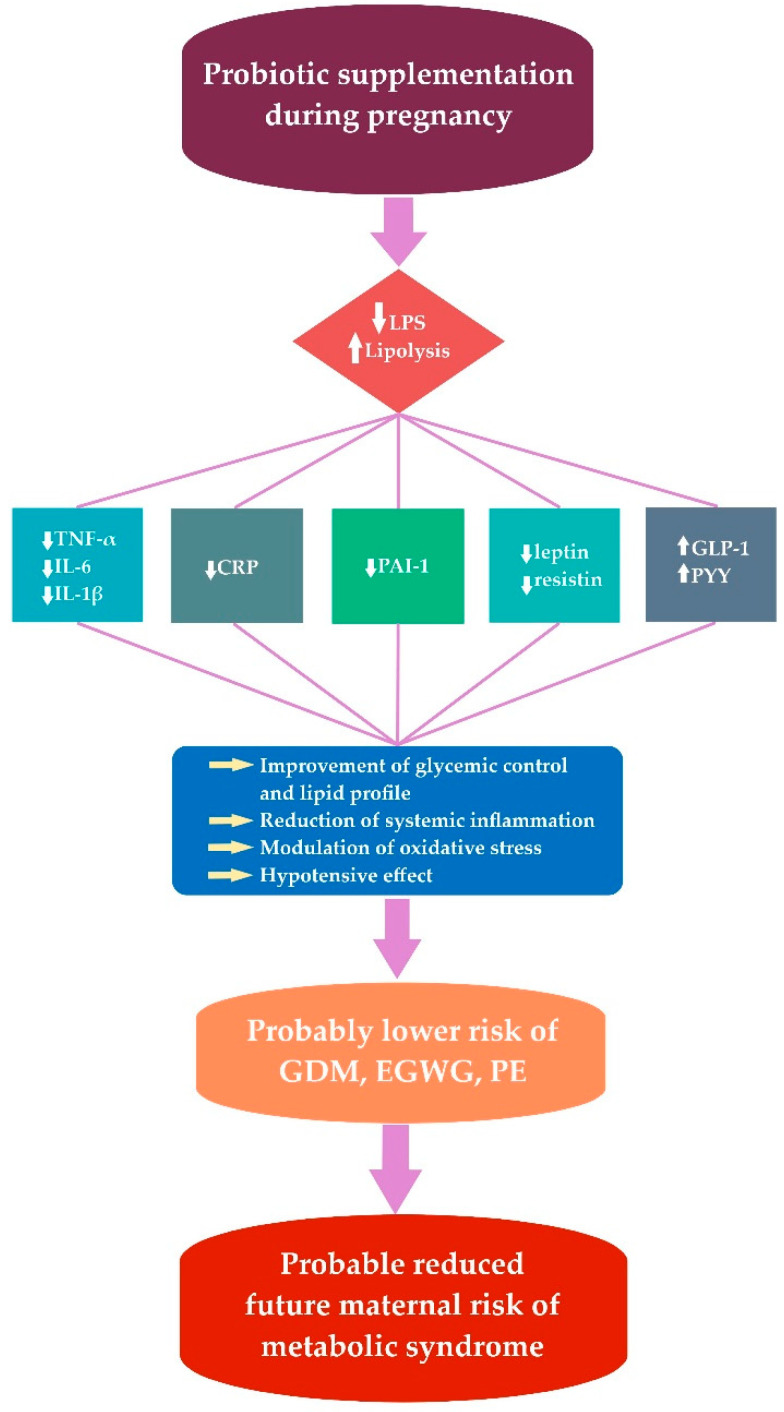
The effect of probiotic supplementation during pregnancy. LPS (lipopolysaccharide); TNF-α (tumor necrosis factor α); IL-6 (interleukin 6); IL-1β (interleukin 1 beta); CRP (C-reactive protein); PAI-1 (plasminogen activator inhibitor-1); GLP-1 (glucagon-like peptide 1); PYY (peptide YY); GDM (gestational diabetes mellitus); EGWG (excessive gestational weight gain); PE (preeclampsia).

Even modest reductions in maternal glucose levels in nondiabetic women, especially those at high risk of GDM, may decrease the risk of developing maternal MS in the future. Effective postpartum follow-up of patients diagnosed with GDM is essential since GDM may progress to T2DM and MS, both of which are major public health problems. Pregestational obesity is predictive of progression to MS, while patients with high FPG and insulin requirements during pregnancy are at an increased risk of developing T2DM. Targeting and identifying high-risk individuals might delay and possibly prevent MS and T2DM. Knowledge about eventual interactions between the gut microflora in GDM and the host could be a potential therapeutic approach to improve health outcomes in women with GDM. A synergistic approach involving both probiotic supplementation and lifestyle modification (diet and exercise) may be a new way to prevent the development of MS in women with GDM. Probiotic supplements can help people with metabolic disorders maintain bacterial diversity and homeostasis because certain microorganisms in the gastrointestinal tract can have a positive effect on host metabolism (Figure 1).

At the present time, there is no consensus regarding the effectiveness of symbiotic or probiotic supplements in GDM management due to limited evidence. Further high-quality studies of longer duration are required to determine the optimal dose, safety and the optimal composition of probiotics used in the group of patients at risk of developing MS. Additional research on the efficacy of probiotics for lipid management is also warranted. The development of obesity, NASH and NAFLD may contribute to the onset of MS in the mother in the future.

The occurrence of PE during pregnancy may be an early warning sign of metabolic complications, described in the literature as “a window to future health”. Women who have been diagnosed with metabolic disorders in pregnancy should be monitored in the puerperium and later in life for the development of these disorders. In patients with a history of PE, it is worth recommending a lifestyle change, reduction of excess body weight and regular medical examinations, which can prevent them from developing MS. It can be hypothesized that probiotics may reduce the risk of PE by modulating blood pressure and reducing inflammation, and more research is needed to elucidate their mechanisms of action and their safety for mother and child. Attention should be paid to reports on the possibility of increasing the risk of PE by taking probiotics and conducting research with due care.

## Figures and Tables

**Table 1:** The criteria for the diagnosis of MS.

Measure	NCEP ATP3 2005	IDF 2009
Elevated waist circumference	≥88 cm (≥34.6 inches)	≥80 cm (≥31.5 inches)
Elevated triglycerides (TG)	≥150 mg/dL (1.7 mmol/L) or drug treatment for elevated TG	≥150 mg/dL (1.7 mmol/L) or drug treatment for high TG
Reduced high-density lipoprotein (HDL) cholesterol	<50 mg/dL (1.3 mmol/L) or drug treatment for low HDL cholesterol	<50 mg/dL (1.3 mmol/L) or drug treatment for low HDL cholesterol
Elevated blood pressure (BP)	≥130 mmHg systolic BP or ≥85 mmHg diastolic BP or drug treatment for hypertension	≥130 mmHg systolic BP or ≥85 mmHg diastolic BP or drug treatment for hypertension
Elevated fasting glucose	≥100 mg/dL (≥5.6 mmol/L) or drug treatment for elevated blood glucose	≥100 mg/dL (≥5.6 mmol/L) or diagnosed diabetes

**Table 2:** The use of probiotics in the prevention of metabolic disorders in pregnant women.

Reference	Strain	Dosage	Treatment Duration	Population	Results
Kijmanawat et al. (2019) [7]	*Lactobacillus acidophilus* and *Bifidobacterium bifidum*	10^9^ CFU/capsule	4 weeks	30 patients with GDM	- decreased fasting plasma glucose (*p* = 0.034), - decreased fasting plasma insulin (*p* = 0.001) - decreased HOMA-IR (*p* = 0.001)
Nabhani et al. (2018) [111]	Synbiotic capsule consisting of *Lactobacillus acidophilus*, *Lactobacillus plantarum*, *Lactobacillus fermentum*, *Lactobacillus gasseri* with fructooligosaccharide (38.5 mg)	1.5–7.0 × 10^9–10^ CFU/g	6 weeks	45 patients with GDM	- increased HDL-C and TAC levels (*p* < 0.05) - decreased systolic and diastolic blood pressure (*p* < 0.05) - increased LDL-C in the placebo group (*p* < 0.05)
Sahhaf Ebrahimi et al. (2019) [112]	*Lactobacillus acidophilus* and *Bifidobacterium lactis*	10^6^ (300 mg of probiotic yoghurt)	8 weeks	42 patients with GDM	- decreased fasting and post prandial blood glucose (*p* < 0.05) - decrease in the level of HbA1c (*p* < 0.05)
Babadi et al. (2019) [114]	*Lactobacillus acidophilus*, *Lactobacillus casei*, *Bifidobacterium bifidum* and *Lactobacillus fermentum*	2 × 10^9^ CFU/g	6 weeks	24 patients with GDM	- upregulated PPAR-γ (*p* = 0.01), - upregulated TGF-β (*p* = 0.002), - upregulated VEGF (*p* = 0.006), - downregulated TNF-α (*p* = 0.03), - decreased fasting plasma glucose (*p* = 0.02), - decreased serum insulin levels (*p* = 0.001), - decreased insulin resistance (*p* = 0.001), - increased insulin sensitivity ( *p* = 0.001) - decreased TG (*p* = 0.02), - decreased VLDL-C (*p* = 0.02), - decreased total-/HDL-C ratio (*p* = 0.006), - increased HDL-C (*p* = 0.03), - reduction in plasma MDA (*p* < 0.001), - elevation in plasma NO (*p* = 0.01), - elevation in total antioxidant capacity (*p* = 0.01)
Pellonperä et al. (2019) [119]	*Lactobacillus rhamnosus* and *Bifidobacterium animalis ssp. lactis*	10^10^ CFU/capsule	throughout the pregnancy, and until 6 months postpartum	439 overweight or obese pregnant women	- no differences in the maternal pregnancy outcomes (*p* > 0.05), - no change in the glucose, insulin, or HOMA2-IR (*p* > 0.11)
Callaway et al. (2019) [120]	*Lactobacillus rhamnosus* and *Bifidobacterium animalis subspecies lactis*	10^9^ CFU/capsule	throughout pregnancy from the first half of the second trimester	207 overweight and obese women prevent GDM	- no effect of probiotics on carbohydrate metabolism
Badehnoosh et al. (2018) [134]	*Lactobacillus acidophilus*, *Lactobacillus casei* and *Bifidobacterium bifidum*	2 × 10^9^ CFU/g	6 weeks	60 patients with GDM	- decreased fasting plasma glucose (*p* = 0.01) - decreased serum CRP (*p* < 0.001) - decreased plasma MDA (*p* = 0.03) - increased TAC levels (*p* = 0.002) - decreased MDA/TAC ratio (*p* = 0.004)
Okesene-Gafa et al. (2019) [150]	*Lactobacillus rhamnosus GG* and *Bifidobacterium lactis BB12*	6.5 × 10^9^ CFU/capsule	throughout the pregnancy	230 obese pregnant women	- no significant difference in total maternal weight gain
Brantsæter et al. (2011) [175]	*Lactobacillus acidophilus*, *Bifidobacterium lactis* and *Lactobacillus rhamnosus*	10^8^ CFU/mL	the first halfof pregnancy	33,399 primiparous women	- - reduced risk of all PE (OR = 0.80, 95% CI: 0.66, 0.96) - reduced risk of severe PE (OR = 0.61, 95% CI: 0.43, 0.89)
Nordqvist et. al. (2018) [178]	*Lactobacillus acidophilus*, *Bifidobacterium lactis* and *Lactobacillus rhamnosus*	10^8^ CFU/mL	Early pregnancy or late pregnancy	37,050 primiparous women	- reduced risk of PE if taken in late pregnancy (*p* = 0.007) - reduced risk of preterm delivery if taken in early pregnancy (*p* = 0.03)

HOMA-IR (Homeostatic Model Assessment—Insulin Resistance); GDM (gestational diabetes mellitus); HDL-C (high density lipoprotein cholesterol); TAC (total antioxidant capacity); LDL-C (low density lipoprotein cholesterol); PPAR-γ (peroxisome proliferator-activated receptor gamma); TGF-β (transforming growth factor β); VEGF (vascular endothelial growth factor); TNF-α (tumor necrosis factor α); TG (triglycerides); VLDL-C (very-low-density lipoprotein cholesterol); MDA (Malondialdehyde); NO (nitric oxide); CRP (C-reactive protein); PE (preeclampsia).

## Data Availability

Not applicable.
